# Geographic information system (GIS)-based image analysis for assessing growth of *Physarum polycephalum* on a solid medium

**DOI:** 10.1186/s40694-015-0017-z

**Published:** 2015-11-19

**Authors:** Hanh T. M. Tran, Steven L. Stephenson, Jason A. Tullis

**Affiliations:** 1 School of Biotechnology, Ho Chi Minh International University, Ho Chi Minh City, 70000 Vietnam; 2 Department of Biological Sciences, University of Arkansas, Fayetteville, AR 72701 USA; 3 Department of Geosciences, University of Arkansas, Fayetteville, AR 72701 USA

**Keywords:** Myxomycetes, Image, GIS, Plasmodium, Solid culture

## Abstract

**Background:**

The conventional method used to assess growth of the plasmodium of the slime mold *Physarum polycephalum* in solid culture is to measure the extent of plasmodial expansion from the point of inoculation by using a ruler. However, plasmodial growth is usually rather irregular, so the values obtained are not especially accurate. Similar challenges exist in quantification of the growth of a fungal mycelium.

**Results:**

In this paper, we describe a method that uses geographic information system software to obtain highly accurate estimates of plasmodial growth over time. This approach calculates plasmodial area from images obtained at particular intervals following inoculation. In addition, the correlation between plasmodial area and its dry cell weight value was determined. The correlation could be used for biomass estimation without the need of having to terminate the cultures in question.

**Conclusion:**

The method described herein is simple but effective and could also be used for growth measurements of other microorganisms such as fungi on solid media.

## Background

*Physarum polycephalum* Schwein. is a member of the order Physarales of the class Myxomycetes, a group of fungus-like eukaryotic organisms commonly known as slime molds. Like all other members of this group, the life cycle of *P. polycephalum* is characterized by a distinctive multinucleate trophic (feeding) stage called a plasmodium. The plasmodium of *P. polycephalum* occurs on decaying plant material and the fruiting bodies of wood-decaying fungi in nature and has a bright yellow color [1]. The rapid rate of growth, the absence of cell walls and the ease with which the plasmodium of *P. polycephalum* can be cultured have caused it to be widely used in cell biology and behavioral research.

Research that involves a determination of the growth of *P. polycephalum* growth on a solid medium is commonly carried out by measuring the extent of plasmodial expansion from the point of inoculation by using a ruler [2–6]. Although this is a convenient method that requires very little effort, plasmodial growth is often irregular in shape, which makes any determination of actual size rather challenging. Less commonly, the plasmodium in a particular culture is collected and dried for determination of plasmodial growth by weighing the dry material on a small balance. The value obtained in such a manner is referred to the dry cell weight (DCW) of the culture. This method is more accurate than a measurement taken with a ruler, but the cultures must be terminated in order to collect the total biomass of the plasmodium [7].

As a general rule, geographic information system (GIS) software has been used in science, engineering, and business applications at spatial scales linked to common methods of locational observation (e.g., global positioning systems as well as airborne and satellite-based imaging). However, extension of GIS to finer scales has produced innovations such as 3D topographic analysis of primate teeth (e.g., Zuccotti et al. [8]). In a rare microbiological application, Yang et al. [9] used GIS for measuring fungal growth. The mycelial cultures of the fungi being cultured were photographed and fungal growth was determined on the basis of the proportion of the pixel count of the mycelium divided by the sum of the pixel count of the mycelium and substrate. Our comparable approach, also based on pixel counts, represents a new application of GIS to study the growth of a plasmodium. The relatively precise aerial estimates (i.e., when compared with the use of a ruler) are facilitated by (a) digital camera capture of fine spatial resolution 2D images of plasmodia and (b) GIS-based extraction of areal extents of visually unique patterns in the imagery.

Originally based on the work of Ball and Hall [10], ISODATA clustering is commonly available in GIS software and used to enable classification of (or categorical labeling of pixels in) imagery acquired from various cameras and other types of sensors. ISODATA is well suited to cluster (or group) pixels of various colors found in a typical digital camera-captured image of a Petri dish. For example, the various intensities of the yellow color of the plasmodium, the clear raw substrate, the plastic walls of the dish, and the background materials are each candidates for a cluster, depending on the parameterization of ISODATA. The proportion of yellow plasmodium pixels, relative to the total number of pixels in the Petri dish, can be multiplied by the area of the Petri dish to obtain a systematic area of the plasmodium at the time the photo is acquired. Moreover, this approach is especially useful for any project with the goal of tracking the growth of a particular plasmodium over time or for comparing plasmodial growth between different cultures.

The objectives of the project reported herein were first to evaluate the feasibility of using GIS software as a new approach for measuring plasmodial growth and then to determine the correlation (if any) between the pixel areas of plasmodia and their dry cell weight (DCW) increments.

## Results and discussion

### Images of plasmodial growth profile

**Fig. 1:**
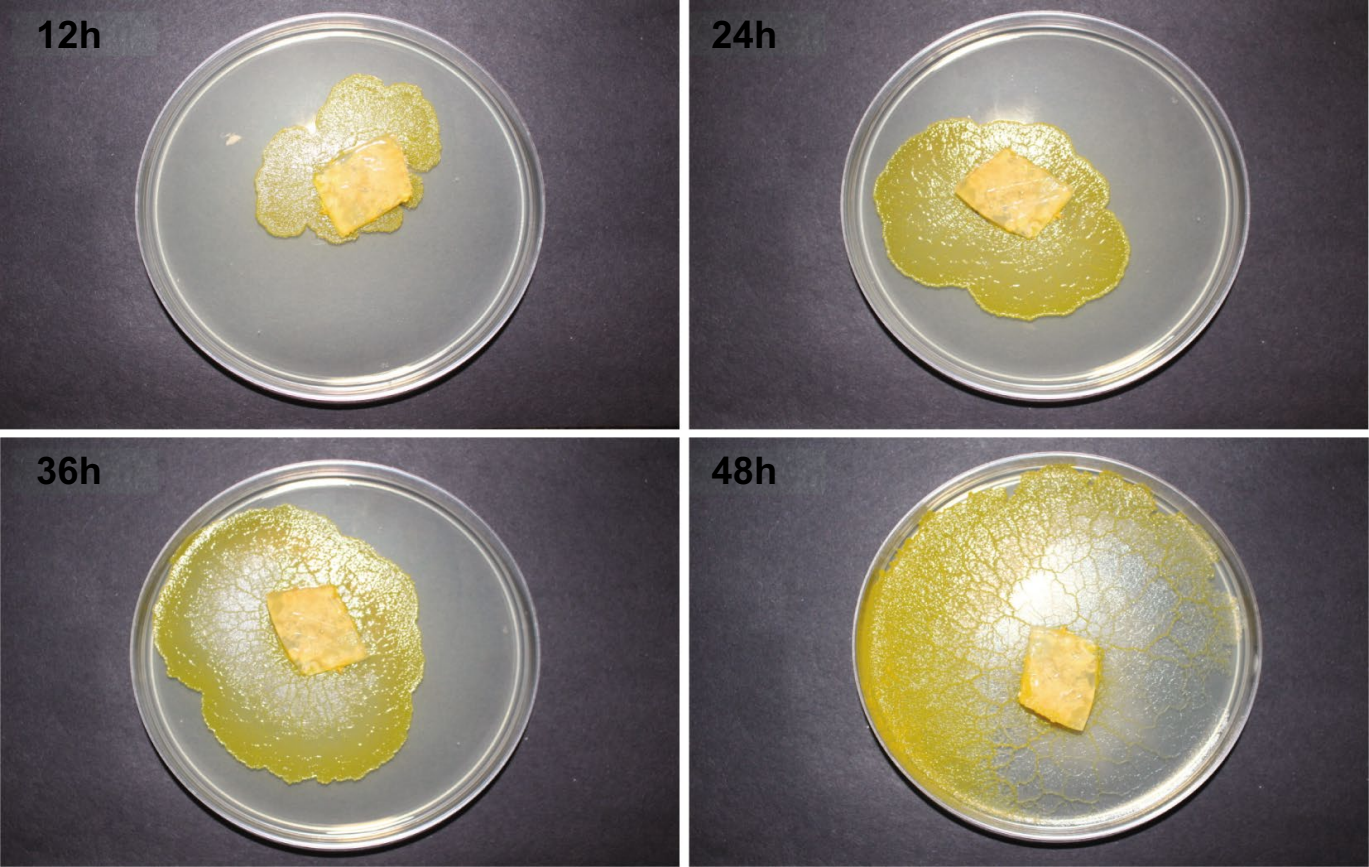
Images of *P. polycephalum* plasmodia taken at different times after inoculation from the same starting culture

*Physarum polycephalum* was grown in nutrient agar medium culture plates. Each plate contained 25 mL of medium. Inoculum was added to the center of the plate. It can be noticed that the plasmodial extensions were irregular and when the plasmodia migrated away from the points of inoculation, they formed slime tracks, which were colorless. This can be observed from the 36 and 48 h images (Fig. 1).

After 48 h, the plasmodia began clumping together and formed thicker biomass (image not shown). More discussion about this phenomenon is provided below.

### GIS analysis of plasmodial images

Eight plasmodial cultures were prepared using the same inoculum size and medium composition as described above. Every 12 h, images of the cultures were obtained and plasmodium was collected from two randomly selected plates for later comparison of growth area and DCW analysis. The original images of the plasmodial culture used for GIS analysis were taken using a Canon EOS 100D camera. All of the images had the same resolution (1920 × 1280 pixels) and were saved as JPEG files.

The plasmodial images were analyzed using Esri’s ArcGIS 10.2 for Desktop GIS software with the Spatial Analyst (image analysis) extension enabled. An iterator (for automated looping through plasmodium images) was set up using a graphic block programming capability (ModelBuilder) native to ArcGIS. When executed, the iterator transformed each original JPEG plasmodium image into a grayscale image, with each brightness value representing a unique cluster of similar pixels. Through ISODATA, a signature of each cluster (or unique group of pixels in the plasmodium photo) was determined and reported as (a) mean blue, green, and red brightness values, and (b) a three-band variance–covariance matrix. Configured to search for 100 unique clusters, ISODATA used an iterative process to calculate up to 100 of these unique signatures [10].

**Fig. 2:**
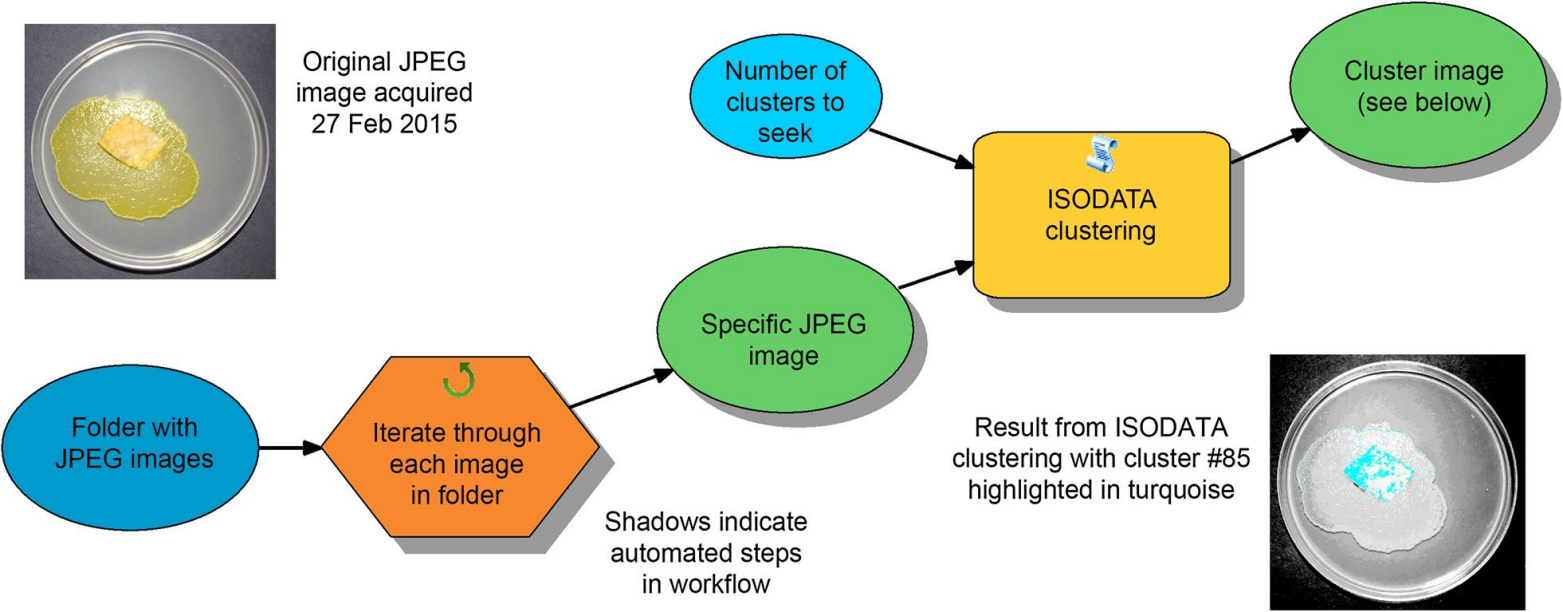
ArcGIS 10.2 for Desktop ModelBuilder workflow including an iterator to automate the processing of multiple plasmodial images. Each input image file is classified into as many as 99 clusters. Individual clusters (e.g., #85 highlighted here in *turquoise*) can be selected for expert determination as to whether they represent a plasmodium or some other component of the image

With the ISODATA signatures used for reference, a Maximum Likelihood Classifier (MLC) was applied to sort each pixel of the image into a cluster. This produced a grayscale image showing the patterns for the various components (e.g., Petri dish, plasmodium, etc.) but with each brightness value representing a unique cluster or color subcomponent of the image (Fig. 2). Using the diameter of the Petri dish in pixels and a spreadsheet, the pixel counts for a selection of plasmodium clusters were obtained and converted to a value for area.

While comparable to the technique used by Yang et al. [7] in terms of counting target pixels, this technique relies upon selection of appropriate target (plasmodium) clusters instead of extraction of pixels based on a blue channel threshold. This technique also eliminates the need for masking out background components of the image, since these simply become clusters that will not be selected.

Many software tools can be adapted to achieve the same image processing results described above, and a number of studies have demonstrated successful image processing approaches for observing spatial and temporal patterns of plasmodia [11–14]. The GIS-based image analysis approach offers an expanding array of tools and intuitive interfaces, workflows history (provenance), and excellent documentation influenced by the current proliferation of multidisciplinary image-producing technologies.

### Pixel area and DCW profiles of plasmodial cultures

**Fig. 3:**
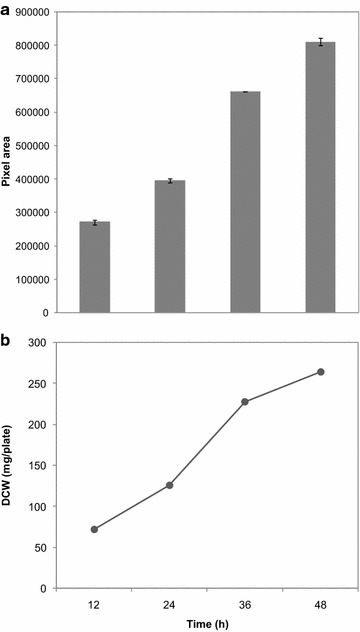
Total pixel areas (**a**) and corresponding DCWs (mg/plate) (**b**) of *P. polycephalum* plasmodium at different times after inoculation. Note: each plate contained 25 mL nutrient medium

As indicated in Fig. 3, both pixel area and DCW of the culture increased exponentially over time. It should be noted that after 48 h, the plasmodium began clumping together and formed areas with a thicker biomass. The DCWs still kept increasing exponentially, but pixel area of the corresponding plasmodium did not follow the same pattern; however, it was still significantly higher than the previous value. Presumably, to avoid plasmodial cluster formation, larger containers would need to be used. However, even with cluster formation it should be possible to extract more precise geometric estimates of plasmodial growth from 2D digital photographs and GIS. For example, varying degrees of plasmodial thickness will naturally register as different shades of yellow, and therefore may be grouped into different ISODATA clusters. Using an average estimate of plasmodial thickness for given shades of yellow, it should be possible to estimate plasmodial volume from the respective 2D areas. Future research should examine this approach to determine if improved correlations between GIS-assisted and DCW-based measurements.

### Correlation between plasmodial culture area and DCW

**Fig. 4:**
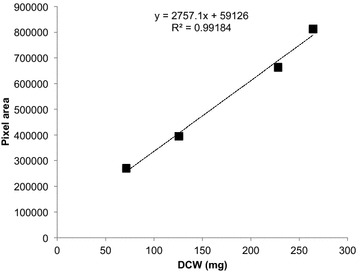
Correlation between pixel area and DCW of the culture

The relationship between plasmodial culture area and DCW was calculated as

$$y = 2757.1x + 59126 \,\{ 70 < x < 270\}$$

where $y$ is pixel area and $x$ is DCW (mg) (Fig. 4). With R^2^ at 0.99 (over the range observed from approximately 70–270 mg DCW), this relationship is very promising.

As mentioned previously, however, a high degree of accuracy with respect to data on pixel area would be obtained only if the plasmodium does not form clusters.

Determining the correlation between the pixel area of a plasmodium and its DCW provides researchers an additional option for assessing plasmodial growth and the actual product (biomass) being produced over time without terminating the cultures. For instance, the lipid content of the *P. polycephalum* plasmodium was found to be approximately 10 % of the DCW [7]. In the most commonly used method to determine the lipid amount of a culture, the plasmodium is collected and DCW is determined to derive an estimation of the lipid amount. However, in the method described herein, which uses GIS software, DCW can be calculated from pixel area and the amount of lipid determined accordingly.

## Conclusions

A new approach for myxomycete plasmodial growth measurement was developed using GIS software. This approach allows a more accurate assessment of the growth of the plasmodium over time than can be obtained with the use of traditional methods. Moreover, the correlation between pixel area of a plasmodial image and its DCW can be used for biomass estimation with a high degree of accuracy.

## Methods

### *Physarum polycephalum*

The strain of *P. polycephalum* used in the present study was obtained as a sclerotium from Carolina Biological Supply Company (Burlington, North Carolina).

### Medium used

The medium used for activation of the sclerotium was water agar (1.0 L of water agar containing 20.0 g of agar and 1000 mL of distilled water), whereas the medium used for plasmodial growth was nutrient agar (1.0 L of the nutrient agar containing 100 mL of a basal salt solution, 5.0 g of glucose [Difco], 2.5 g of yeast extract [Difco], 20.0 g of agar, and 900 mL of distilled water adjusted to pH 5.5). The basal salt solution contained 29.78 g of citric acid, 33.10 g of K_2_HPO_4_, 2.50 g of NaCl, 1.00 g of MgSO_4_·7H2O, 0.50 g of CaCl_2_·2H_2_O, and 1000 mL distilled water.

### Plasmodial activation

The plasmodium of *P. polycephalum* was activated by placing the sclerotium on the surface of a water agar plate (10 cm diameter). Once activated, sterile oat flakes were added on the actively growing plasmodium and the latter incubated in the dark for 1 day. A plug of agar (ca 2 cm^2^) bearing a portion of the active plasmodium growing in association with the oat flakes was used as an inoculum and transferred to larger plates (14 cm in diameter) containing different types of media. Plates were incubated in the dark at room temperature (ca. 22–23 ℃).

### Acquiring images of cultures

A digital image of each culture was obtained every 12 h. In order to maintain the same distance, lightning and size/aspect in all of the images, the camera used to take the images was attached to a small tripod and the latter remained untouched during the entire period the experiment was carried out except when each new image was acquired. Black paper was used as the background to achieve maximum contrast with the color of the culture dish and plasmodium. The position at which the cultures were placed was predetermined and marked on the paper. It should be noted here that to avoid light reflection, paper with a rough surface was selected instead of one with a shiny/smooth surface. In addition, to minimize color bias, culture information was recorded separately on a removable label and the latter was not included in the image obtained of the culture.

### Determination of dry cell weight

Fresh biomass of *P. polycephalum* was collected from each culture and lyophilized (Labconco, freeze-zone 6) to a constant weight and weighed on an analytical balance (AB104-S, Switzerland). This value represented the dry cell weight (DCW).

### Experimental statistics

All the experiments were carried out in duplicate. The figures included in this paper are derived from the mean values (±standard errors) obtained from both sets of cultures.
